# Organ-organ communication: The liver's perspective

**DOI:** 10.7150/thno.55795

**Published:** 2021-01-16

**Authors:** Fei Wang, Kwok-Fai So, Jia Xiao, Hua Wang

**Affiliations:** 1 Division of Gastroenterology, Seventh Affiliated Hospital of Sun Yat-sen University, Shenzhen, China.; 2 GMH Institute of CNS Regeneration, Guangdong Medical Key Laboratory of Brain Function and Diseases, Jinan University, Guangzhou, China.; 3 Laboratory of Neuroendocrinology, Fujian Key Laboratory of Developmental and Neurobiology, School of Life Sciences, Fujian Normal University, Fuzhou, China.; 4 Clinical Medicine Research Institute and Department of Interventional Surgery, First Affiliated Hospital of Jinan University, Guangzhou, China.; 5 Department of Oncology, the First Affiliated Hospital of Anhui Medical University, Anhui Medical University, Hefei, China.; 6 Inflammation and Immune Mediated Disease Laboratory of Anhui Province; School of Pharmacy, Anhui Medical University, Hefei, China.

**Keywords:** Liver, Organ communication, Hepatokine, Cytokine, Disease mechanism.

## Abstract

Communication between organs participates in most physiological and pathological events. Owing to the importance of precise coordination among the liver and virtually all organs in the body for the maintenance of homeostasis, many hepatic disorders originate from impaired organ-organ communication, resulting in concomitant pathological phenotypes of distant organs. Hepatokines are proteins that are predominantly secreted from the liver, and many hepatokines and several signaling proteins have been linked to diseases of other organs, such as the heart, muscle, bone, and eyes. Although liver-centered interorgan communication has been proposed in both basic and clinical studies, to date, the regulatory mechanisms of hepatokine production, secretion, and reciprocation with signaling factors from other organs are obscure. Whether other hormones and cytokines are involved in such communication also warrants investigation. Herein, we summarize the current knowledge of organ-organ communication phenotypes in a variety of diseases and the possible involvement of hepatokines and/or other important signaling factors. This provides novel insight into the underlying roles and mechanisms of liver-originated signal transduction and, more importantly, the understanding of disease in an integrative view.

## Introduction

Over the past 30 years, we have witnessed advances in the understanding of how organs and tissues communicate under healthy and pathologic conditions by secreting proteins, lipids, metabolites, and small noncoding RNAs. Now, it is considered that most tissues are able to communicate with local and distant tissues/organs in an autocrine/paracrine/endocrine manner. Interorgan and intertissue communication is increasingly recognized as a critical way to maintain homeostasis and disease adaptation 1. Furthermore, molecular biology has provided a toolkit to identify the functions of circulating proteins, such as hormones and cytokines, as communication messengers. Indeed, many new communication proteins are yet to be discovered that will widen our knowledge of organ-organ communication loops and provide potential novel strategies for treating some degenerative diseases 2.

The liver performs a number of essential functions related to detoxification, nutrient storage, digestion, metabolism, and immunity. Liver diseases, including viral hepatitis, nonalcoholic fatty liver disease (NAFLD), alcoholic liver disease (ALD), autoimmune hepatitis (AIH), fibrosis, and end-stage liver disease, account for approximately 2 million deaths per year worldwide 3. Clinically, both acute and chronic liver diseases have evident extrahepatic complications, which in turn determine the disease progression and efficacy of therapy, forming 'organ-organ communication' 4, 5. Thus, understanding the interorgan communicating mechanisms, especially the molecular mediators therein is important for the development of targeted therapies.

## Communicating molecules derived from the liver

Although first identified in metabolic diseases (e.g., NAFLD and type 2 diabetes), signaling proteins exclusively or predominantly secreted from the liver termed 'hepatokines' are now known to directly affect other diseases. Hepatocytes secrete more than 560 types of hepatokines, many of which regulate metabolic and inflammatory diseases in the liver or at distant organs (e.g. the endocrine roles of hepatokines in bone and heart tissues) through circulation delivery 6. Under extreme challenges, such as long-term starvation or overnutrition, the liver may secrete hepatokines to influence energy homeostasis and inflammation. Indeed, if the liver is unable to fulfill this process, the corresponding disease develops, such as steatosis from impaired hepatic insulin-sensitizing substance production 7.

### Fibroblast growth factor-21

Fibroblast growth factor-21 (FGF-21) is a hormone predominantly secreted by the liver to regulate glucose metabolism in adipocytes in addition to insulin activity. It has been recognized as a therapeutic target of obesity and diabetes 8. Injection of recombinant FGF-21 in obese mice successfully ameliorated hepatic steatosis, glucose intolerance, and insulin resistance through increased insulin production and secretion 9. Recent studies also identified FGF-21 as a biomarker for a variety of human diseases, including metabolic disorders 10, drug-induced liver injury 11, hypertension 12, and mitochondrial disease 13. Currently, the findings of high FGF-21 levels in those patients conflict with the beneficial effects of exogenous FGF-21 administration in patients with obesity and those with diabetes, which might be attributed to possible FGF-21 resistance, suggesting that high levels of FGF-21 are a compensation mechanism in response to metabolic stress in those patients.

### Adropin

Adropin is a recently identified small peptide for the maintenance of energy homeostasis and insulin resistance. Its plasma level is positively correlated with nutritional status, that is, upregulated upon feeding and downregulated with fasting 14. Patients with NAFLD, obesity, and cardiovascular disease are reported to have low levels of adropin, indicating that this peptide is associated with metabolic disorders 15. In a NAFLD mouse model, overexpression of adropin significantly improved insulin resistance and glucose intolerance 16. Moreover, muscle insulin signaling was also improved through the amelioration of mitochondrial dysfunction 17. When adropin was knocked out, mice exhibited increased adiposity, dyslipidemia and whole-body insulin resistance 18. A recent study unveiled the regulatory effects of adropin on fuel substrate utilization in the heart, probably *via* a novel G protein coupled receptor (GPCR)-mitogen-activated protein kinase (MAPK)-signaling pathway 19. Adropin protein expression falls progressively with advancing age in the human peripheral vasculature. Restoration of adropin may facilitate the improvement in endothelial function via increased nitric oxide bioavailability 20.

### Angiotensinogen

The renin-angiotensin system (RAS) is pivotal to the regulation of blood pressure and water/sodium metabolism. As a hepatokine, angiotensinogen (AGT) is the only precursor of all angiotensin peptides. Accumulating evidence shows that AGT is not only a passive substrate of the RAS, but also plays a critical role in the pathogenesis of obesity and atherosclerosis. Transgenic human AGT in mice showed augmented atherosclerosis when fed a high-fat diet for 14 weeks 21. Hepatocyte-specific knockout of AGT in mice resulted in lowered body weight and ameliorated liver steatosis/atherosclerosis after high-fat diet feeding 22. Indeed, deficiency of AGT could reduce basal plasma AGT levels and blood pressure and induce renal medial hyperplasia 23. Since adipose tissue is the only extrahepatic source to have an impact on plasma AGT concentration, the sole role of adipocyte-derived AGT and the communication between the liver and adipose tissue during disease progression have attracted mass attention in recent years. Adipocyte-specific deficiency of AGT decreased systolic blood pressure and prevented obesity-induced hypertension in mice 24, 25.

### Fetuins

**Figure 1:**
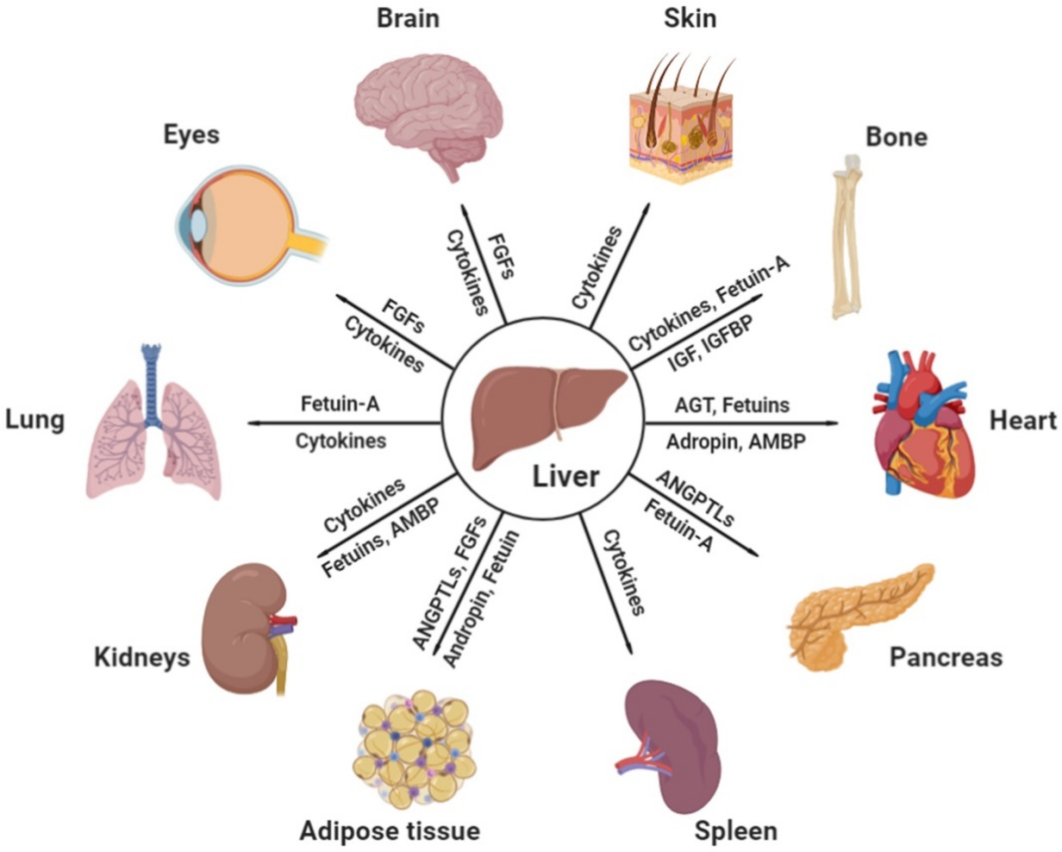
**Effective communicating molecules, mostly hepatokines and cytokines, from the liver to other major distant organs under physiological and pathological conditions.** AGT, angiotensinogen; AMBP, alpha-1-microglobulin; ANGPTL, angiopoietin-like protein; FGF, fibroblast growth factor; IGF, insulin growth factor; IGFBP, insulin growth factor binding protein (Created with BioRender.com).

Both fetuins-A and-B are important hepatokines in human metabolism regulation. Fetuin-A was first identified as a natural inhibitor of the insulin receptor tyrosine kinase in liver and skeletal muscle. Deficiency of fetuin-A in mice exhibited improved phenotypes of insulin signaling 26. Human studies also confirmed that, independent of adiposity, circulating fetuin‑A levels positively correlated with high liver fat content, early atherosclerosis, and metabolic syndrome 27, 28. In addition, fetuin-A induces insulin resistance and inflammation via multiple mechanisms, including the activation of the extracellular signal regulated-kinase-nuclear factor kappaB (ERK-NF-κB) pathway 29, promoting saturated fatty acid-induced activation of toll-like receptor 4 30, inducing proinflammatory cytokine production in monocytes and adipocytes 31, and inhibiting insulin-sensitizing adiponectin production 32. Recent articles also described the critical roles of fetuin-A in diseases of other organs, such as the heart, kidneys, and bone, which will be discussed in the following sections. Like fetuin-A, fetuin-B is also a liver-derived plasma protein. It is increased in patients with type 2 diabetes and impairs insulin sensitivity in myotubes and hepatocytes 33. The current knowledge of the effects of liver-secreted cytokines and hepatokines on distant organs under physiological and pathological conditions is illustrated in Figure 1 and Table 1.

## Communications between the liver and other major organs

### Gut-liver-brain axis

The concept of the gut-liver-brain axis was first reported in the study of lipid metabolism and glucose synthesis by Wang, *et al.* in 2008 34. To date, this axis is the most well-studied interorgan pathway for the liver. Bile acids (BAs), the metabolites of cholesterol, have long been considered to play an essential role in lipid digestion and fat-soluble vitamin absorption. Two typical bile acid receptors, farnesoid X receptor (FXR) and Takeda G protein-coupled receptor 5 (TGR5), are abundantly expressed not only in the enterohepatic circulation but also in the brain 35, 36. Thus, studies have focused on the central nervous system activities in which BAs are involved 37, 38. A recent study discussed the relationship among bile acids, intestinal microbiota and Alzheimer's disease in which the interaction between bile acids and intestinal microbiota influenced the function of the brain 39. Mechanistically, intestine-restricted agonism of FXR induced TGF5 activity, promoted secretion of FGF-15/21 to improve insulin resistance and promote white adipose tissue browning in mice 40. This provides a theoretical basis for the direct or indirect risk factors of cognitive decline diseases caused by cholesterol metabolism disorder, including cholestatic liver disease, dyslipidemia, fatty liver disease, cardiovascular disease and diabetes.

Hepatic encephalopathy (HE) manifests as brain dysfunction caused by liver diseases, and portosystemic shunting is one of the late complications of cirrhosis 41. HE occurs due to different pathogeneses in which hyperammonemia and gut-derived toxins (e.g. indoles, oxindoles and endotoxins) have been considered to be the major pathological causes. Ammonia and the proinflammatory environment are mainly derived from intestinal microbiota, especially *Enterobacteriaceae* and *Streptococcaceae*, and subsequently, ammonia is transported to the liver and metabolized 42, 43. The critical involvement of Kupffer cells in HE pathogenesis was revealed in an ammonium acetate-challenged model where specific deletion of TLR9 in macrophages ameliorated brain edema and lymphocyte cytokine production 44. Since the ammonia levels in chronic liver failure do not reliably correlate with HE severity and many HE cases are caused in an ammonia-independent manner, treating HE through microbiota modulation (e.g. lactulose and rifaximin) has up to now been considered a first line of intervention 45.

The gut-liver-brain axis is also involved in the regulation of glucose metabolism. Intestinal lipids activate the intestinal vagus nerve and transduce the signal to the brain, which then regulates the synthesis of glycogen 34. Additionally, it was reported that cholecystokinin, protein kinase A, and protein kinase C activation were all involved in regulating the synthesis of glycogen through the gut-liver-brain axis 46-48. In alcoholic liver disease, new evidence shows that coexistent depression and other psychiatric conditions can have a role in ALD progression, because only ~15-20% alcohol use disorder patients develop ALD 49. Physiologically, a complex consisting of one of the FGF-21 receptors (FGFR1c, FGFR2c or FGFR3c) and a single-pass transmembrane protein, β-Klotho, renders FGF-21 signals able to regulate circadian behavior by acting on the hypothalamus and the dorsal vagal complex of the hindbrain 50. FGF-21 also mediates endocrine control of simple sugar intake and sweet taste preference by the liver 51. Physical exercise is known to improve both hepatic and brain health, which involves an increased secretion of FGF-21 and IGF-1 (insulin-like growth factor 1), leading to improved cognitive function and brain metabolism 52.

### Liver-adipose tissue communication

Expandability of adipose tissue directly contributes to the progression of metabolic liver diseases, such as NAFLD and ALD 53. When adipose tissue reaches the upper limit of lipid storage capacity, excessive lipids will be redirected to other organs, most notably the liver. This process warrants a precise modulation of extracellular matrix remodeling and neovascularization of adipocytes. Once deregulated, hypertrophic subcutaneous adipocytes induce lipolysis and to secrete more pro-inflammatory cytokines (e.g. TNF-α and IL-1β) which promote local and hepatic insulin resistance and inflammation 54, 55. In opposite, a hyperplastic expansion, where the number of adipocytes increases, is thought to be linked to the maintenance of normal insulin sensitivity 56. We recently reported that chronic-binge ethanol consumption significantly reduced both the mass and adipocyte size of white adipose tissues and increased the secretion of pro-lipolysis and inflammatory molecules (e.g. adipose triglyceride lipase, TNF-α, and IL-6), which aggravated the severity of ALD in a mice model 57.

FGF-21 exerts profound effects on energy expenditure and whole-body glucose metabolism largely in the adipose tissue via an insulin-independent manner. Adipose tissue production of adiponectin is increased in FGF21 transgenic mice and markedly inhibited in FGF21-null mice, possibly because of the impaired 'autocrine loop' between FGF21 and PPARγ 58. Similarly, serum adropin level is higher in mice fed with HFD than mice with chow diet. Adropin knock-out mice exhibit a 50%-increase in increase in adiposity while injection of bioactive peptide adropin effectively improves insulin resistance and metabolic flexibility in HFD-fed mice 17, 18. Fetuin-A mRNA expression is elevated in NASH compared with steatosis patients 59. Interestingly, accumulated fetuin A protein (but no mRNA can be detected) is observed in subcutaneous adipose tissue of obese than that of non-obese human, implying that fetuin A potentially acts as a hepatokine taken up by adipose tissue directly from the liver or circulation rather than local production 60. Angiopoietin-like protein-3 and -4 (ANGPTL3/4) are circulating hepatokines acting on lipoprotein lipase (LPL) inhibition to regulate plasma lipid levels 61. ANGPTL8/betatrophin, or lipasin, is expressed primarily in liver and visceral adipose tissue and can promote hepatosteatosis and plasma triglyceride levels in humans. Its action on LPL activation needs the co-inhibition of ANGPTL3/4 to enhance triglyceride uptake 62. Retinol-binding protein 4 (RBP4) is primarily secreted from the liver but also released from adipose tissue as an adipokine. Serum and hepatic RBP4 levels are associated with NAFLD to NASH progression in clinical trials 63, 64. Adipocyte-derived RBP4 further contributes to insulin resistance and obesity 65. Hepatocyte-specific knockdown of *rbp4* (without influence in white adipose tissue) improved insulin sensitivity and glucose metabolism in diabetic mice 66. Recent study found that increased serum concentrations of ANGPTL1, 2, 8, and FGF21, as well as decreased levels of ANGPTL4 and adiponectin were associated with NAFLD-associated HCC 67.

### Liver-pancreas communication

Liver and pancreas play complementary roles in glucose metabolism and homeostasis. Interlobular and total pancreatic fat are both positively related to NAFLD activity score and total pancreatic fat is a significant predictor for the presence of NAFLD in patients 68. Another study also indicates that fatty liver plays a prognostic role in acute pancreatitis 69. Excessive alcohol ingestion often leads to evident pancreatic injury. Both a single occasion of binge drinking and chronic alcohol consumption can increase the risk of an acute attack of pancreatitis. In the US, approximately 1 in 3 cases of acute pancreatitis and 4 in 10 causes of chronic pancreatitis are caused by alcohol, respectively 70, 71. Metabolites of alcohol (e.g. acetaldehyde) processed by the pancreas damage the acinar cells, promote premature intracellular digestive enzyme activation, and thereby induce the local autodigestive injury 72. Importantly, acute pancreatitis patients with liver cirrhosis have higher inpatient mortality compared to non-cirrhotics, possibly due to complications of cirrhosis and portal hypertension itself during the decompensated stage 73.

In 2013, Yi *et al.,* claimed that ANGPTL8 secreted from the liver controlled pancreatic beta cell proliferation and mass increase 74. However, this observation was quickly challenged and proved to be incorrect by following studies which found that *Angptl8* knockout mice exhibited no abnormalities in terms of glucose homeostasis and/or in beta cell expansion in response to HFD- or insulin receptor antagonist S961-induced insulin resistance 75, and liver-specific overexpression of *Angptl8* did not increase beta cell proliferation in mice 76. Fetuin-A induces chemokine (MCP-1 and IL-8) and cytokine (IL-6) production in human pancreatic pre-adipocytes and adipocytes. It also stimulates IL-1β expression in islet-infiltrating macrophages through TLR4, which reveals the role of fetuin-A-mediated metabolic crosstalk between fatty pancreas and fatty liver 77. Hepatocyte-derived kisspeptin1, under the control of glucagon, will transport to pancreas and binds to abundantly expressed kisspeptin 1 receptor in the beta cells, to inhibit cyclic AMP production and insulin secretion 78. It has been demonstrated that pancreatic FGF21 was a digestive enzyme secretagogue whose physiologic function was to maintain acinar cell proteostasis 79, and restoring pancreatic FGF21 reverses pancreatitis induced by cerulein, alcohol, or endoscopic retrograde cholangiopancreatography 80. However, whether liver-secreted FGF21 participate such process remains largely unknown. Moreover, damaged pancreas may secrete pro-inflammatory cytokines (e.g. TNF-α) to directly attack the liver *per se*.

### Liver-eye communication

Modern clinical investigations suggest that noticing common manifestations in the eyes can help people prevent or address an issue with liver well-being, such as scleral icterus reflecting jaundice and xanthelasma palpebra for possible hepatic steatosis. Supplementation with lutein, a well-recognized eye health-promoting carotenoid, improved oxidative stress and inflammation in the liver and eyes of guinea pigs fed a hypercholesterolemic diet 81. A recent study even found that retinitis pigmentosa-induced oxidative stress in the retina might influence soluble macromolecules exiting the retina or significantly impair the melanopsin system, resulting in chronic circadian desynchronization and weakened systemic antioxidant defense, which ultimately caused hepatic oxidative stress 82. Another interesting study found that bright-light therapy towards the eyes could clinically ameliorate pruritus of cholestasis since this chronic liver disease-related skin disorder was centrally mediated by endogenous opioid peptides that induce disrupted circadian rhythm 83. In a mice HE model, we identified that thioacetamide-challenged liver could release proinflammatory cytokines (TNF-α and IL-6) to cause impairments in the brain and eyes. Amelioration of liver injury significantly improved eyesight and cognition in mice 84. This is in line with a previous clinical report in which a patient with severe acute liver failure had uncontrolled intracranial hypertension and needed a hepatectomy that resulted in stabilization of the systemic and cerebral hemodynamics. The removal of the liver was associated with a sharp and sustained reduction in the circulating proinflammatory cytokine concentration 85. A recent study revealed that FGF-21 administration suppressed retinal neovessel growth and local TNF-α expression in a mouse model, partly through an adiponectin-dependent manner 86. What and how liver-derived hepatokines and cytokines are delivered to the eyes are the most urgent questions to be answered.

### Liver-bone communication

The prevalence of osteopenia/osteoporosis/fractures in chronic liver disease is between 12% and 55%, which is much higher in cholestatic diseases 87. Hepatic osteodystrophy commonly manifests with osteoporosis and osteopenia, while osteomalacia is rare 88. The pathogenic mechanisms for hepatic osteodystrophy are not well defined and are considered to be multifactorial. Impaired IGF-1 secretion from the liver might be one of the causative factors of hepatic osteodystrophy because it is a bone collagen and osteoblast stimulator 89. In addition, increased unconjugated bilirubin can inhibit osteoblast differentiation and proliferation 90. The association between viral hepatitis and bone dysfunction has been established in recent years. For example, untreated hepatitis C virus (HCV) infection is found to have a higher fracture risk 91. Interestingly, except in the African-American population, untreated hepatitis B viral (HBV) infection had no significant difference in the incidence of hip fracture when compared with uninfected controls 92. Low body weight, malnutrition, smoking habits, alcohol consumption, opioids or antidepressive drug use, and hypogonadism will also contribute to viral hepatitis-associated bone disease 93. Similarly, several recent cross-sectional and case-control studies have shown that NAFLD patients have a greater prevalence of decreased BMD than age-, sex-, and body mass index-matched healthy controls 94, 95. Proven links between NAFLD and decreased bone mass include vitamin D insufficiency 96, the growth hormone/IGF-I axis 97, TNF-α 98, the receptor activator of NF-κB (RANK)/osteoprotegerin pathway 99, fetuin-A 100, osteopontin 101, and adiponectin 102. In addition, chronic inflammation and irisin from osteoblasts have been proposed as mediators of mutual interactions among the skeleton, fatty tissue, and liver 103. Patients with ALD often have accompanying decreased vitamin D levels, which is primarily attributed to reduced hepatic 25-hydroxylase activity, malabsorption, irregular feeding habits and lack of sun exposure 104. Zinc deficiency is also described among chronic alcoholics. Since zinc stimulates osteoblastic bone formation and mineralization, its deficiency decreases bone mass and is associated with an increased risk of fracture 105. Blockade of liver-derived IGFBP-1 inhibits bone loss mediated by FGF-21 without inhibiting insulin sensitivity. *In vivo* administration of IGFBP-1 promotes osteoclast differentiation and bone resorption 106.

### Liver-heart communication

Manifestations from the liver are very common in cardiac dysfunction or congestive hepatopathy. The pathological features of chronic cardiogenic liver disease include liver congestion, sinusoidal dilatation, necrosis and fibrosis in the central lobule of the liver 107. Patients with acute heart failure and/or hypovolemia often develop hypoxic hepatitis, which has similar clinical manifestations as acute viral hepatitis. In addition, cirrhosis can further lead to the redistribution of circulating blood volume, as well as abnormal cardiac contractility and electrophysiology, which in turn leads to cirrhotic cardiomyopathy 108. Half of the patients with cirrhosis had prolonged QT (Q wave to T wave) intervals, which might be an important factor to evaluate patient survival and prognosis. In addition, the plasma membrane fluidity, calcium channel disorders, and many factors such as cytokines, nitric oxide and endocannabinoids, also participate in the pathophysiological processes of cirrhotic cardiomyopathy 109.

Several systematic conditions affect the liver and heart simultaneously. NAFLD and cardiovascular diseases are usually related to metabolic disorders, such as obesity and diabetes. A multicenter community-based longitudinal cohort study has shown that NAFLD is an independent risk factor for subclinical myocardial remodeling and dysfunction after the assessment of body mass index (BMI) and ventricular activation time (VAT). Patients with NAFLD demonstrated cardiac diastolic dysfunction and had higher left ventricular (LV) mass, LV end-diastolic volume, and LV relative wall thickness than non-NAFLD participants 110. A NAFLD risk allele *TM6SF2* (transmembrane 6 superfamily member 2; rs58542926 c.449 C>T, p.Glu167Lys) was found to be associated with dyslipidemia and cardiovascular risk, primarily due to impaired lipidation and reduced secretion of VLDL (very-low-density lipoprotein) particles from the liver 111. The heart and liver are also common targets of alcohol abuse, which has been proved to damage the heart structure to a greater extent than to the liver 112. The liver-heart inflammatory axis has a pivotal pathological role in the development of hepatic cardiomyopathy. Cannabinoid-2 receptor activation markedly improved hepatic/myocardial inflammation, decreased serum TNF-α level, and cardiac dysfunction, underlining the importance of inflammatory mediators in the pathology of this disease 113. Hepatocyte-specific knockout of IL-6 was sufficient to block aging-induced cardiac arrhythmia in a fruit fly model 114. In addition to the characterized linking function of adropin 19 and fetuin 33, a recent study reported that alpha-1-microglobulin (AMBP) exacerbated inflammation and disturbed hepatic fibrotic repair after myocardial infarction through activating Akt, NF-κB, and ERK signaling and promoting macrophage migration and polarization 115. Another hepatokine, hepcidin, has been proven to regulate macrophage inflammation and arterial stiffness, thus promoting the development of atherosclerosis 116, 117. Upregulated fetuin B aggravates myocardial ischemia/reperfusion injury through inhibiting insulin signaling in diabetic mice 118. Gut microbial metabolism of choline results in hepatic production of trimethylamine-*N*-oxide (TMAO), which exacerbates atherosclerosis via promoting forward cholesterol transport 119, 120. In an acute myocardial infarction mice model, it was revealed that supplementation of IL-22 markedly prevented left ventricular dysfunction and heart failure via liver-derived STAT3-FGF21 production, in which hepatocyte-specific knockout of STAT3 or FGF21 blocked such alleviation of IL-22 121.

### Liver-kidney communication

Hepatorenal syndrome is a serious complication that can lead to death. It is caused by severe liver diseases (e.g., acute liver failure and cirrhosis) and induce progressive kidney failure 122. Apart from that, a number of liver disorders may also involve renal damage, such as viral hepatitis, ALD, NAFLD, and Wilson disease 123. Two major renal diseases associated with chronic HBV are membranous nephropathy and polyarteritis nodosa. Membranoproliferative glomerulopathy, IgA nephropathy, and amyloidosis may also occur 124. In a recent retrospective cohort analysis involving 56,448 HCV patients in the United States, these patients had a 27% increased risk of chronic kidney diseases (e.g., membranoproliferative glomerulonephritis, and cryoglobulinemia) compared with patients without HCV in which the viral protein deposits may mediate the interorgan pathogenesis 125. Patients with ALD have a tendency to exhibit impaired sodium and fluid handling, as well as severe alterations in the body's acid-base balance, which directly influence the homeostasis of the kidneys 126. Intuitively, alcoholics should have a higher risk of chronic kidney disease. However, one meta-analysis comprising 20 studies with a total of 292,431 patients found a decreased risk or no risk of kidney disease in heavy alcohol consumers 127. This result was confirmed by other similar studies showing that the incidence of kidney disease is comparable or even lower in heavier drinkers (>210 g/wk of ethanol) than in moderate drinkers (70-210 g/wk of ethanol) 128, 129. Emerging evidence suggests that NAFLD aggravates insulin resistance and atherogenic dyslipidemia, promoting the release of proinflammatory, prooxidant, profibrogenic, and procoagulant mediators from the liver or kidneys to induce renal injury 130. Interestingly, although vitamin D deficiency has been linked to the pathogenesis and severity of NAFLD and renal diseases, benefits of vitamin D supplementation remain controversial, which may require higher dose treatment or vitamin D receptor agonists (e.g., paricalcitol) to be overcome 131. Although the detailed mechanism remains controversial, the application of a recombinant hepatokine protein, AMBP, has been proven to be effective for ischemic acute kidney injury and chronic renal disorder therapies 132. In an angiotensin II-induced vascular dysfunction mouse model, replenishment of FGF-21 directly regulates the multi-organ crosstalk between liver, adipose tissue, kidney, and blood vessels to improve hypertension and renal injury 12. As a key regulator of the entry of iron into the circulation, hepcidin is also believed to contribute to increased iron sequestration and subsequent anemia in patients with chronic kidney disease 133. A very recent study found that IL-6-mediated hepatocyte production was the primary source of plasma and urine neutrophil gelatinase-associated lipocalin during acute kidney injury 134.

### Liver-skin communication

The notion of 'skin reflects liver health' have been recognized for a long time. For example, jaundice is usually recognizable when serum bilirubin levels are high. Whether the excessive bilirubin is conjugated or unconjugated implies whether the cause is prehepatic, intrahepatic, or posthepatic 135. When the neutralizing function of the liver is disrupted, deposition of toxins and filtered bile salts in the skin causes irritation and itching 136. Another well-known skin sign of severe liver disorder is spider angiomas induced by elevated estrogen levels, such as in cirrhosis 137. Recently, interorgan communication between the liver and the skin under pathological conditions has received much attention. Acyl-CoA-binding protein (ACBP) knockout mice display delayed metabolic adaptation to weaning. Further mechanistic studies found that this phenomenon, including hepatic lipid adaptation, was caused by ACBP deficiency in the skin rather than in the liver 138. The association between liver disease and psoriasis has been reported by several studies. For example, studies suggested that psoriasis may be more severe in patients with NAFLD/NASH 139, 140. Mice fed a high-fat diet developed steatohepatitis reminiscent of human NASH. Cotreatment with imiquimod, a commonly used agent for psoriasis-like systemic inflammation models, exacerbated psoriatic phenotypes, including scale formation and acanthosis, through an IL-17A-dependent mechanism 141. Conversely, another study found that a 9-week topical treatment with imiquimod induced typical psoriasiform dermatitis and, surprisingly, moderate portal/periportal hepatitis and liver fibrosis in mice. Livers from psoriatic mice were enriched for macrophages, polymorphonuclear neutrophils, and T cells 142. AIH is often concomitant with extrahepatic autoimmune diseases. The positive association between AIH (particularly type 2 AIH) and vitiligo is well documented. Probable associations are also identified with alopecia areata, psoriasis, and pyoderma gangrenosum, although they were probably underdiagnosed and underreported in patients with AIH 143. IGF-1 released from the liver contributes to skin wound healing. Deficiency of IGF-1 receptor keratinocytes disrupts epidermal homeostasis and stem cell maintenance 144. Of note, all of the organs from liver specific IL-22 transgenic mice had a normal histology except for slightly thicker epidermis and minor inflammation in the skin compared with WT mouse skin 145. However, the exact communicating mechanisms are not well characterized.

### Liver-spleen communication

Liver cirrhosis often leads to splenomegaly and frequently accompanying hypersplenism, which is considered the main cause of cytopenia and thrombocytopenia in those patients 146. Portal vein blockade is widely considered the initial cause of splenomegaly during liver cirrhosis. Conversely, since the spleen is the body's largest immune organ and plays an important role in the production of antigen-specific T cells and numerous cytokines, a recent study demonstrated the effects of splenic immunity on liver fibrosis and reported that after liver injury, T helper (Th)1 cells suppressed liver fibrosis, while Th2-dominant splenic lymphocytes migrated into the liver and promoted liver fibrosis by shifting the cytokine balance towards Th2 dominance 147. Splenectomy also accelerates hepatic regeneration in cirrhotic animals and patients with cirrhosis and inhibits the formation of hepatic fibrosis 148, 149. Potential activation of splenic macrophages and subsequent transforming growth factor (TGF)-β1 production might be the major mechanisms. After splenectomy, the capacity of liver regeneration is improved by the decrease in TGF-β1 and the increase in hepatocyte growth factor. Splenectomy significantly increased the hepatic accumulation of Ly-6C(lo) monocytes or macrophages in a thioacetamide-induced murine model of liver cirrhosis with hypersplenism, implicating a role for the splenic control of hepatic monocyte or macrophage phenotypes 150. Moreover, splenectomy suppressed regulatory T cell and hepatic fibrogenesis levels, implicating a role for the splenic modulation of the liver via alterations in T cell subsets 151. Splenectomy improved liver function in patients with liver cirrhosis, especially with large spleen and lower alanine aminotransferase (ALT) levels, and stem cell therapy efficacy 152.

Several reports have discussed the messengers linking the liver and the spleen. An early interesting study found that the modification of inflammatory mediator generation by splenectomy or inhibition of Kupffer cell function may be beneficial for the prevention of endotoxin-induced liver injury after partial hepatectomy 153. Another study demonstrated that cytokine expression in the spleen affects the progression of liver cirrhosis through liver-spleen crosstalk. Expression of TGF-β1 and cytokines such as IL-6 produced by macrophages could affect the progression of liver fibrosis and regeneration in patients with liver cirrhosis 154. Foxp3-expressing CD25^+^CD4^+^ Treg cells in the spleen lead to tolerance of immunity after liver transplantation 155. Enlargement of spleen is commonly seen in patients with of NAFLD, which is caused by disrupted iron metabolism, low vitamin D status, and increased cytokine communication (e.g. IL-6). Moreover, inflamed the visceral adipose tissue during NAFLD may also induce splenic injury via adipokines and cytokines 156.

### Liver-lung communication

From a physiological point of view, the lung and the liver are closely coordinated. When liver function is perturbed, the inactivation of enterogenous pulmonary vasodilators by hepatocytes is lost, contributing to the accumulation of vasodilators 157. At the same time, the increase in nonenteric vasodilators leads to the abnormal expansion of pulmonary vascular and gas exchange disorders, which further triggers hypoxemia and a series of other pathophysiological changes and clinical symptoms. These symptoms are clinically known as hepatopulmonary syndrome, which is common in patients with cirrhosis 158. Tuberculosis (TB) is a chronic respiratory infection caused by *Mycobacterium tuberculosis*, which invades multiple organs and systems, with lung infections being the most common. Hepatic manifestation is seen in up to 50-80% of TB cases. Coinfection with HCV greatly promotes the occurrence of liver damage in patients with pulmonary tuberculosis, possibly because of decreased erythrocytes and T lymphocytes 159. It should be noted that anti-TB drugs, including the three major first-line drugs isoniazid, rifampicin, and pyrazinamide, are the most common adverse events necessitating therapy interruption and causes of DILI, which has a general incidence of 5.3% 160. Acute lung injury/acute respiratory distress syndrome (ALI/ARDS) is an inflammatory response regulated by multiple inflammatory mediators and cytokines. Activation of the liver X receptor alpha (LXRα) pathway has been shown to alleviate LPS-induced ALI by the natural product platycodin D 161. Decreased fetuin A predicts increased disease activity in obstructive lung disease, Crohn's disease, and ulcerative colitis 162. Serum fetuin A promotes Lewis lung carcinoma in a calcium-dependent fashion 163. By using omics methods, it was identified that circulating fetuin B and andropin levels could be used as biomarkers for lung function evaluation in COPD (chronic obstructive pulmonary disease) and myeloperoxidase anti-neutrophil cytoplasm autoantibody-associated lung injury patients, respectively 164, 165. Other communicating proteins are yet to be discovered for the liver-lung communication.

## Conclusions

**Figure 2:**
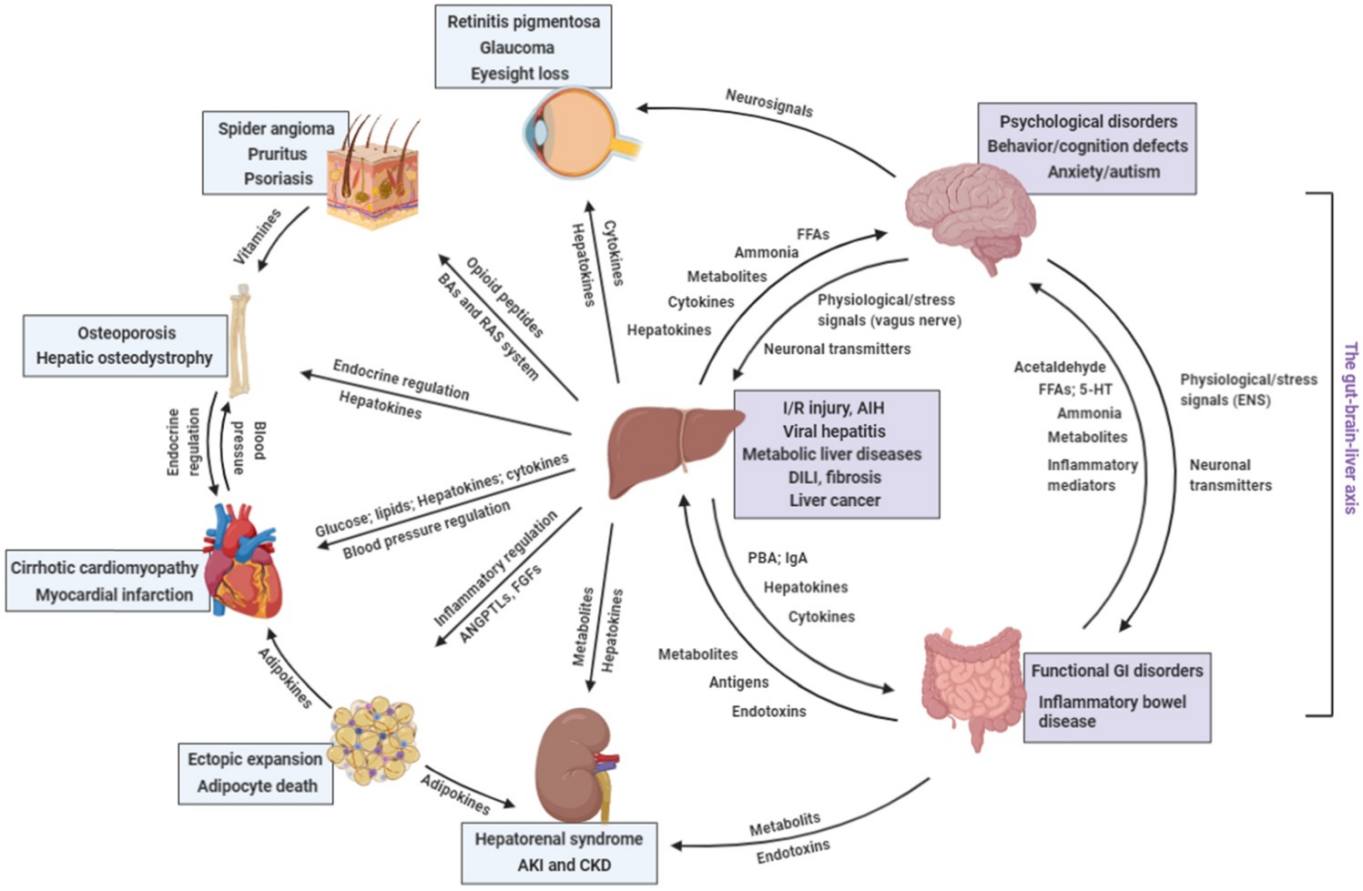
**Distant communicating mechanisms, including neuronal, hormonal, metabolic, and other factors, between major organs under pathological conditions.** 5-HT, 5-hydroxytryptamine; AIH, auto-immune hepatitis; ANGPTL, angiopoietin-like protein; DILI, drug-induced liver injury; FFA, free fatty acids; GI, gastrointestinal; IgA, immunoglobulin A; I/R injury, ischemia/reperfusion injury; PBA, primary bile acids; RAS, renin-angiotensin system (Created with BioRender.com).

Keeping a stable internal environment requires precise regulation of whole-body homeostasis in which organ-organ communication plays critical roles (Figure 2 and Table 2). Since the liver is responsible for a variety of physiological processes, disruptions of homeostatic balance warrant a rapid and well-regulated response from it, in most cases, via paracrine and endocrine signaling molecules, to rebalance itself and distant organs. Importantly, new study has revealed that NAFLD had higher overall risk of incident cancers than obesity without NAFLD, possibly because hepatic ectopic fat act as a paracrine source for cancer development in the liver, gastrointestinal tract and uterus 166. The past decade has witnessed the discovery of a set of hepatokines in many physiological and pathological conditions, in particular, metabolic liver diseases 167. Indeed, other common and rare liver diseases also require communication from the liver to other organs to alleviate pathological events such as inflammation and cell death and to promote local tissue regeneration. Although the functions of several hepatokines and cytokines in organ-organ communications have been preliminarily analyzed, most studies only described the phenotypic relationships between the liver and other organs under different conditions, with the mystery of the direct communicating mediators unanswered. Hence, a future challenge will be to focus on the signals and to elucidate the modulatory mechanisms of liver-generated molecules in disease progression and communication. Specifically, (1) although substantial progress has been made in understanding disease-controlled production of hepatokines, little is known about the inductive mechanism of transcriptional reprogramming, protein translation, modification, and secretion of hepatokines, particularly through the ER and Golgi, by pathological processes; (2) feedback signaling from distant organs that are induced by hepatokines and the reciprocation between hepatokines and other 'organokines' are poorly recognized. Several proteins (e.g. FGF21 and soluble epidermal growth factor receptor) can be secreted from the liver and other organs, which act as hepatokine, adipokine, and/or myokine under different circumstances 168, 169. How these signals are spatio-temporally regulated to maintain functional interorgan communication is largely unknown, which needs cutting-edge techniques, such as specific cell type-transgenic method and single-cell sequencing, to answer; (3) whether different liver diseases (e.g., viral hepatitis and metabolic liver diseases) share similar hepatokine secretion profiles needs further investigation. That is, from a translational perspective, searching a single or set of hepatokines as biomarkers for certain diseases is critical for clinical applications. For metabolic liver diseases, one of the central problems is that they rarely induce specific symptoms and diagnosis is frequently incidental. Recent studies using multiomics approach identified that hepatokine tsukushi was a potential blood biomarker and drug treatment target of NAFLD and subsequent atherosclerosis 170, 171. Indeed, such findings need to be confirmed in large cohorts of corresponding patients (e.g. the pilot clinical findings of FGF21 and LECT2 in diabetic patients) 172, 173; (4) Through the use of advanced mass spectrometry "omics" approaches, we now recognized that liver functions as an endocrine organ by secreting a wide array of hormones (e.g. IGF-1) and noncoding RNAs (miR-9 and -375 for pancreatic insulin regulation), in addition to hepatokines and cytokines, that exert powerful effects on metabolic processes both in the liver and in peripheral tissues 174. Future studies that interrogate the hepatocyte or liver secretomes using multiomics approaches are well positioned to identify novel communicating 'messengers'. Addressing these challenges will be of major interest for the understanding of liver-centered organ-organ communication, as well as the development of rational therapeutic strategies for certain diseases.

## Figures and Tables

**Table 1:** Identified key hepatokines linking hepatic and non-hepatic disorders

Hepatokine	Organ sources	Key functions	Involved diseases	Refs
FGF-21	Mostly liver, also thyroid and pancreas	Reverses hepatic steatosis and increases energy expenditure; Immuno-regulation; Blood pressure and hydration controls	Metabolic disease, DILI, cancers, mitochondrial disease	8, 9
Adropin	Brain, liver, prostate, testis, stomach	Improves insulin sensitivity and energy metabolism; Redox control; Cerebellum development	Metabolic disease, I/R injury, CVD, PCOS	14-17
Angiotensinogen	Mostly liver, also adipose tissue, brain, gall bladder, heart, and kidneys	Blood pressure and water/sodium metabolism regulations	Hypertension, atherosclerosis, metabolic disease, renal disease	22-25
ANGPTLs	ANGPTL1, 3, 4, 6, 8: Mostly liver, also adipose tissue and muscle. ANGPTL 2 and 5: Mostly heart, also adipose tissue; ANGPTL 7: eyes	Lipid and energy regulation; Angiogenesis; Inflammatory regulation; Carcinogenesis and inhibition	Metabolic disease, cardiovascular disease, cancer	61, 62, 67
Fetuin-A	Predominantly liver	Induces insulin resistance and inflammation	Metabolic disease, atherosclerosis, CVD, renal dysfunction, cancer, lung disease	27-30, 167
Fetuin-B	Mostly liver, also esophagus and pancreas	Causes glucose intolerance; Essential for fertilization	Metabolic disease, COPD, CVD, renal disease, osteoporosis	33
IGFBP-1	Mostly liver, also placenta and endometrium	Metabolic regulation; Carcinogenesis,	Bone loss, cancer, metabolic disease	106
AMBP	Mostly liver, also gall bladder	Direct heme binding	CVD, renal disease	115, 132
Hepcidin	Mostly liver, also heart	Inhibits iron transport; Regulatory target of interleukins; Strong antimicrobial activity	CVD, renal disease	116, 117, 133
Tsukushi	Mostly liver	Controls energy expenditure via brown fat sympathetic innervation	Metabolic disease	170, 175

AMBP, alpha-1-microglobulin; ANGPTL, angiopoietin-like protein; COPD, chronic obstructive pulmonary disease; CVD, cardiovascular disease; FGF, fibroblast growth factor; IGFBP-1, insulin-like growth factor binding protein 1; I/R injury, ischemia-reperfusion injury; PCOS, polycystic ovary syndrome.

**Table 2:** Involvement of the liver in other organ's disorders

Organ	Disorder	Hepatic manifestation	Possible linking mechanisms	Refs
Brain	Cognitive disorder (e.g. Alzheimer's disease)	Cholestatic liver disease and fatty liver disease	Bile acids	39
	Hepatic encephalopathy	Cirrhosis and liver failure	Ammonia, cytokines, and hepatokines	42, 43, 176, 177
	Circadian behavior disorder	Lipid dysregulation	FGF-21	50, 51
Adipose tissue	Ectopic expansion and adipocyte death	NAFLD and ALD	FGF-21, ANGPTLs, adropin, fetuin-A	17, 57-59, 67
Pancreas	Acute and chronic pancreatitis	NAFLD and ALD	ANGPTLs, fetuin-A, kispeptin	75, 77, 78
Eyes	Retina injury	NAFLD and ALD	Oxidative stress and cytokines	81, 178
	Retinitis pigmentosa	Liver injury	Oxidative stress	82
Bone	Hepatic osteodystrophy	PBC and PSC	IGF-1, bilirubin,	88, 90
	Fracture	Viral hepatitis, NAFLD, and ALD	Hepatokines, hormones, and vitamins	91, 92, 95, 96, 98, 100, 101, 106
Heart	Heart failure and cardiomyopathy	Liver fibrosis/cirrhosis	Cytokines, calcium, NO, and endocannabinoids	107-109
	Diastolic dysfunction	NAFLD	Free fatty acids (?)	110
	Myocardial infarction	Hepatic inflammation and disorders	Hepatokines	115-117
Kidneys	Hepatorenal syndrome	severe liver diseases (e.g. alcoholic cirrhosis, acute liver failure, and cirrhosis)	Cytokines and hepatokines	122
	Chronic kidney diseases	Viral hepatitis, NAFLD, ALD	Viral-protein deposits, cytokines, hepatokines, ion level, excessive IgA loads and ROS	125, 126
Skin	Spider angiomas	Severe liver diseases	Elevated estrogen level	137
	Psoriasis	NAFLD, ALD, and AIH	Cytokines and hepatokines (?)	137, 139, 141-143
Spleen	Splenomegaly and splenic disorders	Cirrhosis	Portal vein blockade, cytokines and TGF signaling	41, 147, 150
Lung	Hepatopulmonary syndrome	Cirrhosis and acute liver failure	Vasodilator secretion (e.g. NO)	158
	Tuberculosis	Viral hepatitis, DILI,	Cytokines	160
	ALI/ARDS	Liver injury	Hepatokines and cytokines	161, 179

AIH, auto-immune hepatitis; ALD, alcoholic liver disease; ALI/ARDS, acute lung injury/acute respiratory distress syndrome; ANGPTL, angiopoietin-like protein; DILI, drug-induced liver injury; FGF, fibroblast growth factor; IGF, insulin growth factor; NAFLD, non-alcoholic liver disease; NO, nitric oxide; PBC, primary biliary cirrhosis; PSC, primary sclerosing cholangitis; ROS, reactive oxygen species; TGF, transforming growth factor.

## Abbreviations

ACBP: Acyl-CoA-binding protein
AGT: angiotensinogen
AIH: autoimmune hepatitis
AKI: acute kidney injury
ALD: alcoholic liver disease
ALT: alanine aminotransferase
BA: bile acid
CKD: chronic kidney disease
ERK: extracellular signal regulated kinase
FGF: fibroblast growth factor
FXR: farnesoid X receptor
GPCR: G protein coupled receptor
HBV: hepatitis B virus
HCV: hepatitis C virus
HE: hepatic encephalopathy
LPL: lipoprotein lipase
LV: left ventricule
LXR: liver X receptor
MAPK: mitogen-activated protein kinase
NAFLD: nonalcoholic fatty liver disease
NF-κB: nuclear factor-kappa B
RANK: receptor activator of NF-κB
RAS: renin-angiotensin system
RBP: retinol-binding protein
TB: tuberculosis
TGF: transforming growth factor
TNF: tumor necrosis factor
